# Case Report: Resuscitation of patient with tumor-induced acute pulmonary embolism by venoarterial extracorporeal membrane oxygenation

**DOI:** 10.3389/fcvm.2024.1322387

**Published:** 2024-02-15

**Authors:** Shuang-Long Zhang, Qi-Feng Zhang, Gang Li, Miao Guo, Xiao-Xia Qi, Xiao-Hui Xing, Zheng Wang

**Affiliations:** ^1^ Department of Critical Care Medicine, Peking University International Hospital, Beijing, China; ^2^ Department of Neurosurgery, Tianjin Medical University General Hospital, Tianjin, China

**Keywords:** renal cell carcinoma, pulmonary embolism, tumor-induced pulmonary, VA-ECMO, resuscitation

## Abstract

**Background:**

Pulmonary embolism is a condition of right cardiac dysfunction due to pulmonary circulation obstruction. Malignant tumor-induced pulmonary embolism, which has a poor therapeutic outcome and a significant impact on hemodynamics, is the cause of sudden death in patients with malignant tumors.

**Case description:**

A 38-year-old female patient, who had a medical history of right renal hamartoma, and right renal space-occupying lesion, was admitted to the hospital. During the procedure to resect the right renal malignancy, the blood pressure and end-tidal carbon dioxide level dropped, and a potential pulmonary embolism was considered as a possibility. After inferior vena cava embolectomy, the hemodynamics in the patient remained unstable. The successful establishment of venoarterial extracorporeal membrane oxygenation (VA-ECMO) resulted in the stabilization of her hemodynamics and ventilation. On Day 2 of VA-ECMO support, her respiration and hemodynamics were relatively stable, and ECMO assistance was successfully terminated following the “pump-controlled retrograde trial off (PCRTO)” test on Day 6. The patient improved gradually after the procedure and was discharged from the hospital after 22 days.

**Conclusion:**

VA-ECMO can be used as a transitional resuscitation technique for patients with massive pulmonary embolism. It is critical for the perfusion of vital organs and can assist with surgical or interventional treatment, lower right heart pressure, and hemodynamic stability. VA-ECMO has a significant impact on patient prognosis and can reduce the mortality rate.

## Introduction

Cases of pulmonary embolism caused by solid tumor detachment are rare, let alone cases that get diagnosed in time after detachment and are successfully resuscitated with timely venoarterial extracorporeal membrane oxygenation (VA-ECMO). Patients with potential tumor-induced pulmonary embolism should be examined as early as possible and emergency diagnosis and treatment plan for pulmonary embolism after embolus detachment should be prepared in advance during surgery, to reduce the occurrence of malignancy-associated sudden death. In this case, transesophageal ultrasound was performed immediately for the patient after the surgery when circulatory instability was discovered. The ultrasound revealed right ventricular enlargement and pulmonary hypertension, combined with the tumor invasion into the inferior vena cava; thus, massive pulmonary embolism caused by tumor embolus detachment was considered. In the past, the hospital was responsible for ECMO in a certain department. In the past two years, the hospital has organized a special ECMO team composed of experienced doctors from intensive care medicine, cardiac surgery, vascular surgery, emergency department and other related departments. The response speed and operation technology have been improved. Therefore, VA-ECMO was performed by a professional team, and the patient was successfully resuscitated.

## Medical history

**Figure 1:**
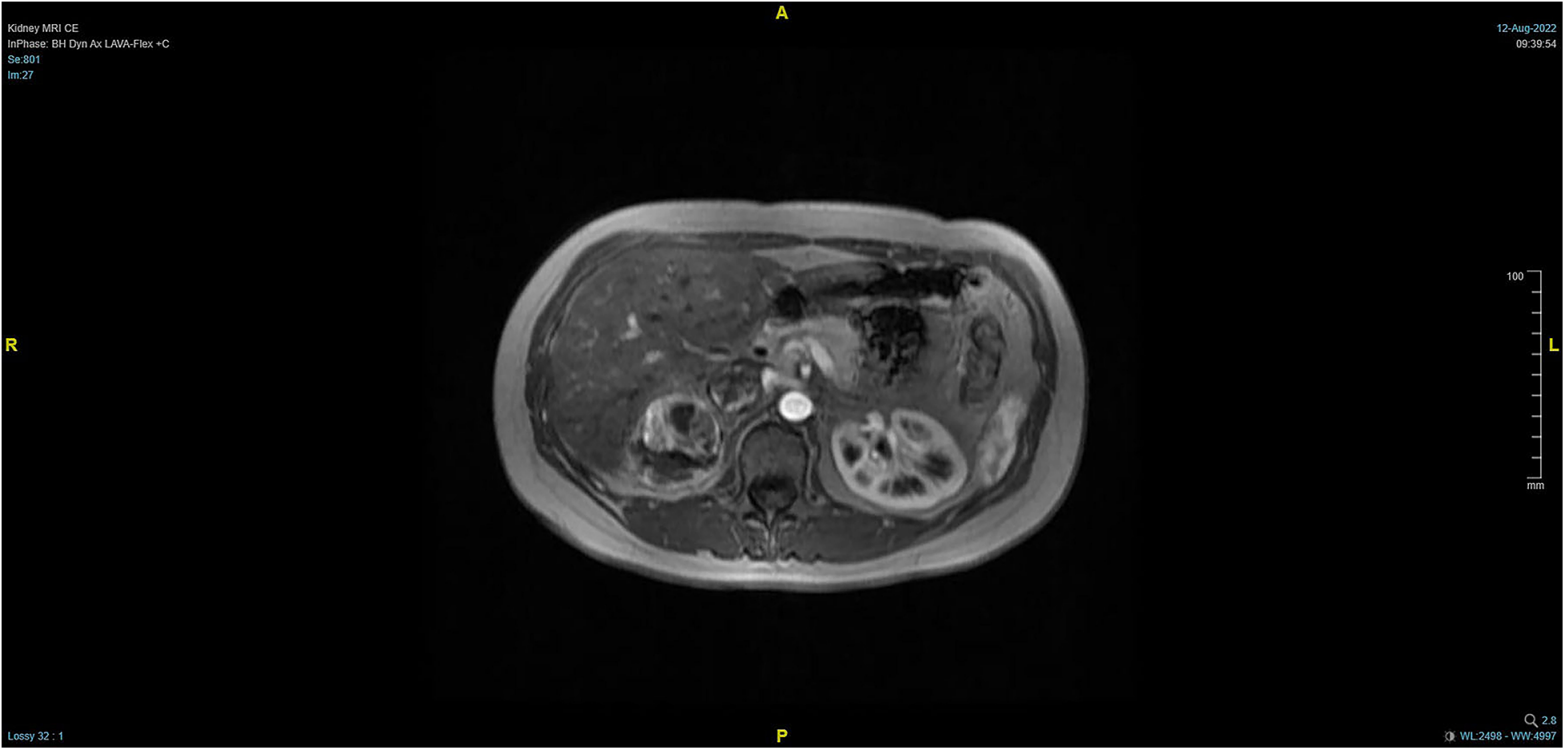
Pre-operative MRI showing that the tumor lesion enlarged upward along the right renal vein into the inferior vena cava (coronal planes).

**Figure 2:**
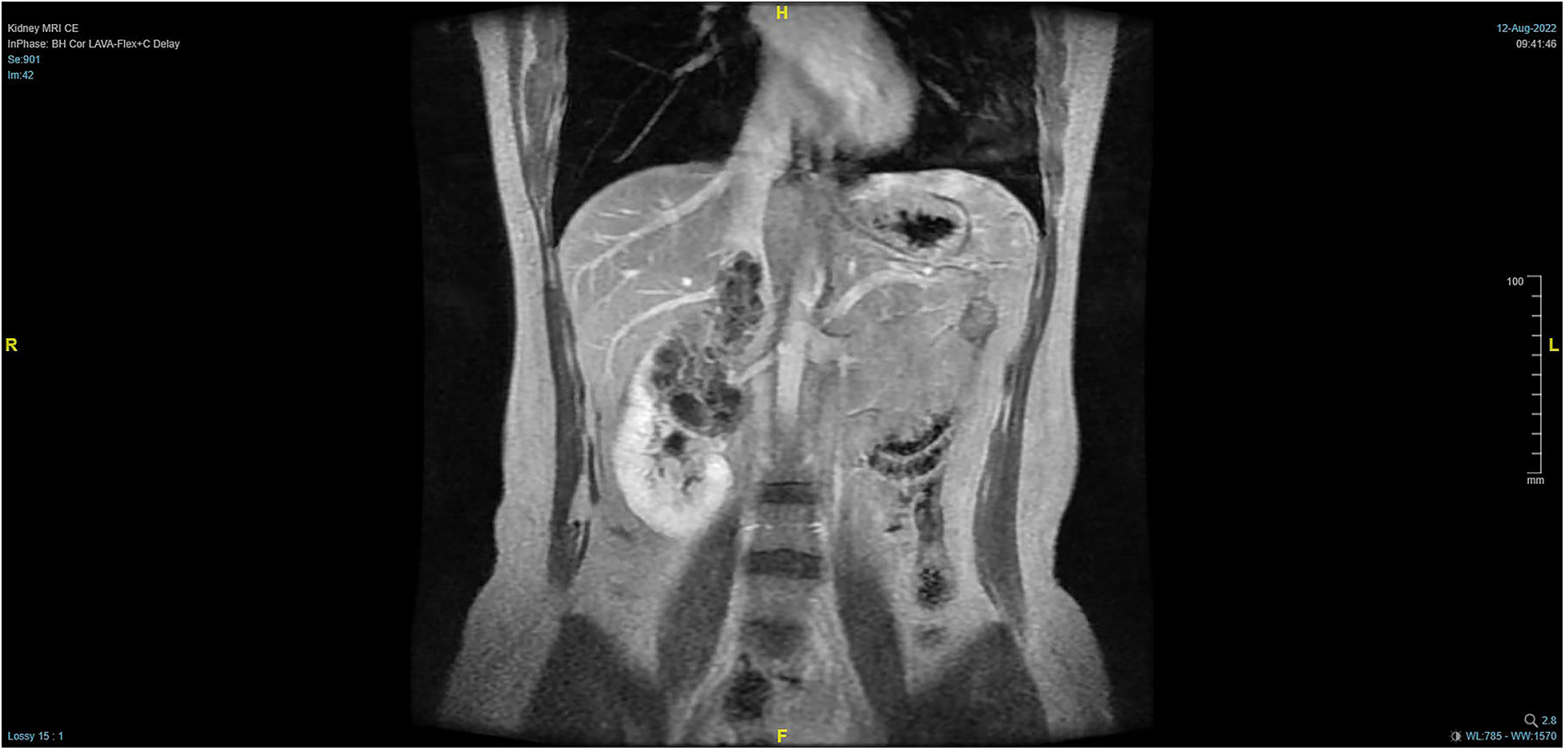
Pre-operative MRI showing that the tumor lesion enlarged upward along the right renal vein into the inferior vena cava (transverse planes).

The patient, a 38-year-old woman, was admitted to the hospital after a physical examination revealed right renal space-occupying lesion for one week. She had no other medical, personal, or family history, other than a history of hamartoma. After admission, the patient underwent the MRI of both kidneys, which revealed an irregularly shaped mass at the upper pole of the right kidney. This mass was characterized by equally short T1 weighted image (T1WI) and T2WI signals, and the signals in the fat-suppression image were not significantly reduced. Multiple cystic components were seen in the mass, some of which were filled with fluid, and the lesion spread into the renal sinus and upward to the inferior vena cava. The right renal veins were not clearly visible. The maximum cross-sectional area of the lesion was about 10.4 × 5.5 cm (coronal). The enhancement scan showed heterogeneity, marked enhancement, separated enhancement, and local clear contour in the delay period. The right kidney was occupied, with multiple cystic variations and hemorrhages in the lesion, and it spread upward along the right renal vein into the inferior vena cava. Malignancy was not ruled out (Figures 1, 2). Urological CT of both kidneys indicated that the right kidney had a space-occupying lesion and that the size and shape of both kidneys were normal.

**Figure 3:**
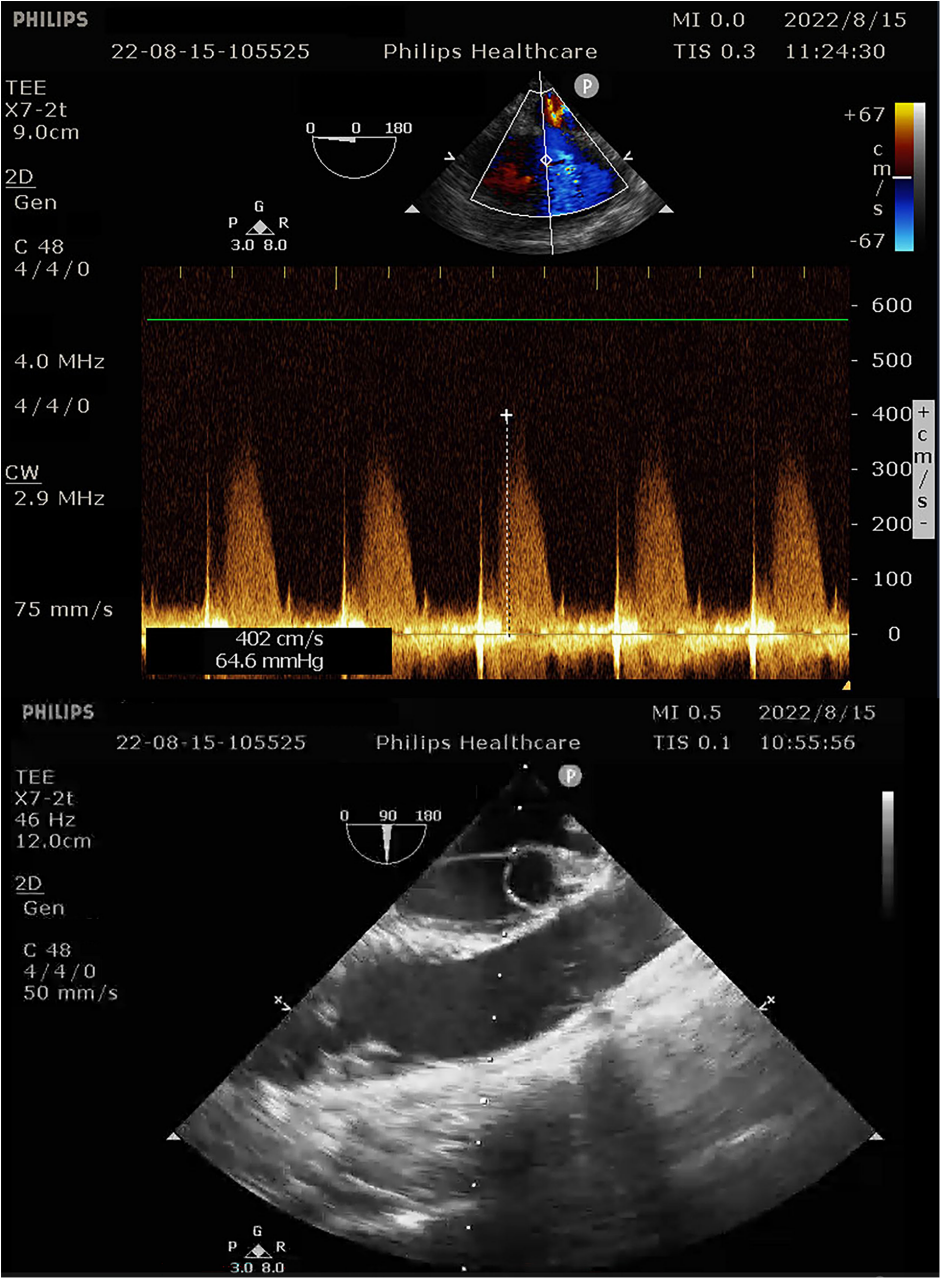
Intra-operative transesophageal ultrasound indicating pulmonary artery pressure of 69 mmHg.

**Figure 4:**
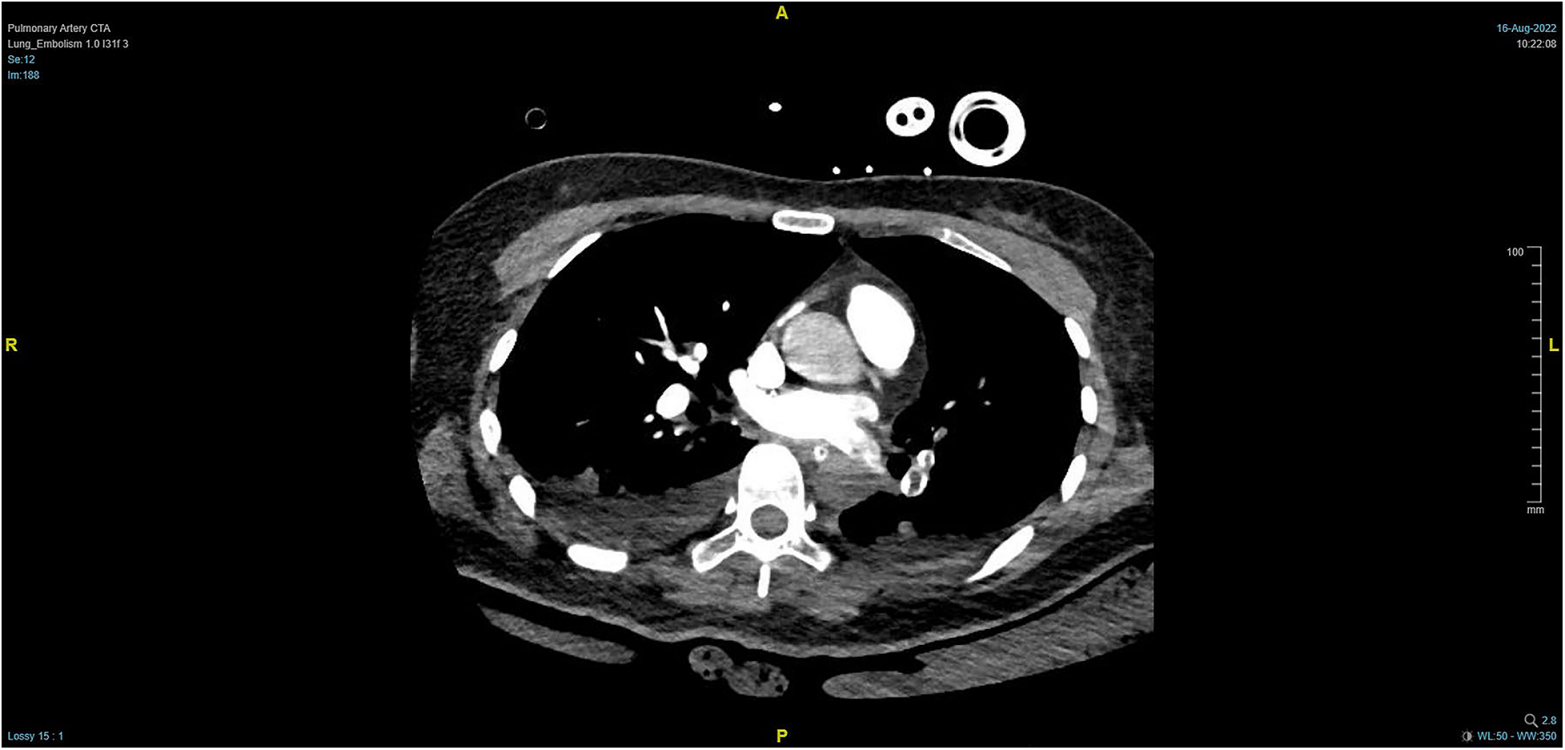
PTCA on Day 2 after operation indicating strip-shaped filling defects in multiple branch arteries of the upper, middle, and lower lobes of the right lung and the lower lobe and lingular segment of upper lobe of the left lung, and multiple pulmonary artery embolisms in both lungs.

**Figure 5:**
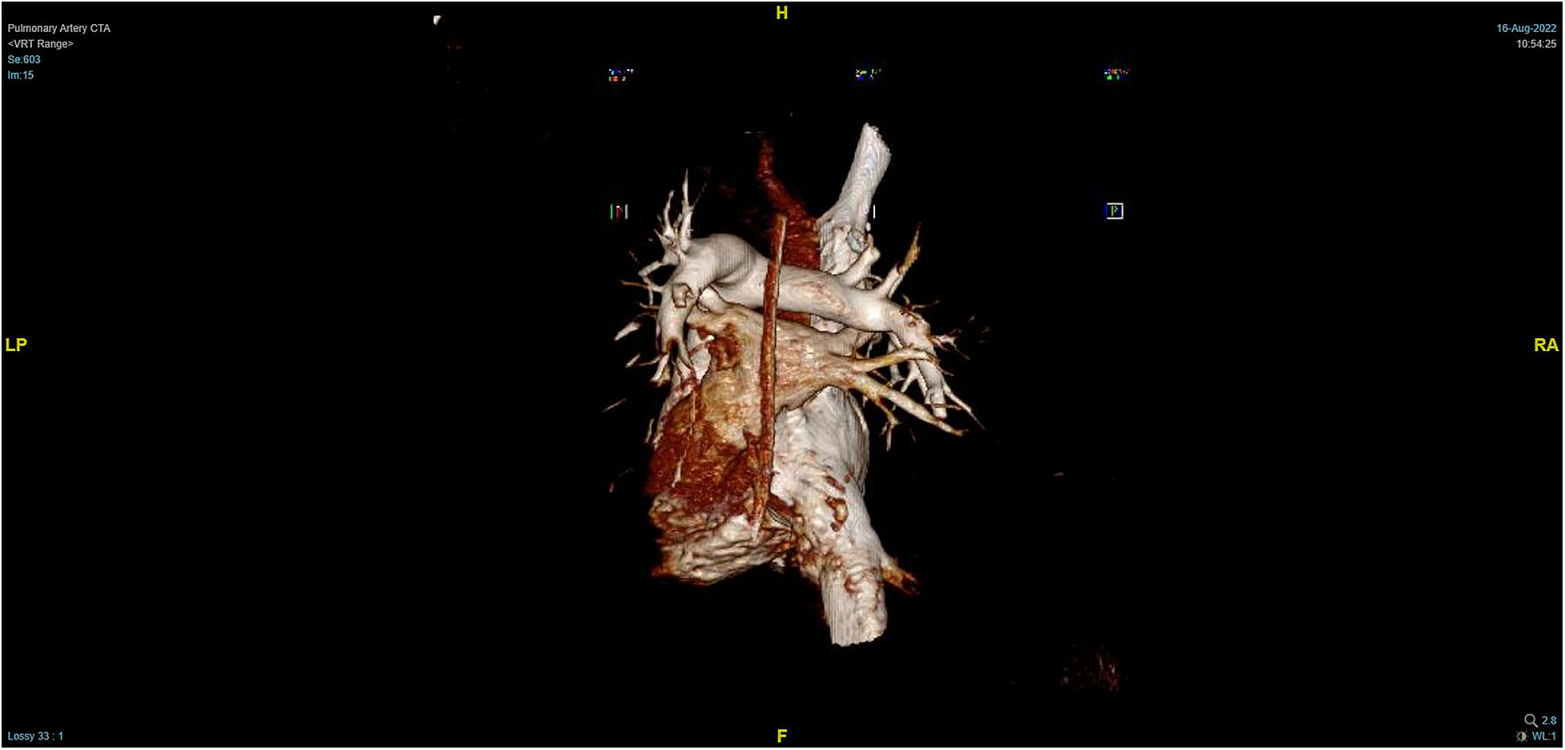
Massive pulmonary artery embolism.

During the procedure to surgically resect the right renal malignancy, the patient experienced decreased blood pressure, low-tidal carbon dioxide and high partial pressure of carbon dioxide in blood gas. After identifying a pulmonary embolism, the hepatobiliary surgeon performed a successful inferior vena cava embolectomy. The hemodynamics was still unstable after the surgery, with high pressure in the pulmonary artery, and the transesophageal ultrasound imaging revealed right ventricular dilation and strain, as shown in Figure 3. The ECMO team determined the indications for VA-ECMO assistance. Then, the right femoral artery was punctured immediately by inserting a 19 F cannula and the femoral vein was punctured by inserting a 21 F cannula. VA-ECMO was successfully established, thereafter, the respiratory status stabilized, and the patient was referred to the Department of Critical Care Medicine. The postoperative pulmonary artery computed tomography angiography (CTA) revealed strip-shaped filled defects in multiple branch arteries of the upper, middle, and lower lobes of the right lung, and the lower lobe and lingular segment of upper lobe of the left lung, and multiple pulmonary artery embolisms in both lungs (Figures 4, 5). Given the extensive embolization of the tumor in this patient, severe pulmonary hypertension may have developed earlier. Therefore, after comprehensive consideration, stronger drugs such as milrinone, ambrisentan, and treprostinil were given to reduce pulmonary artery pressure, along with VA-ECMO support.

**Figure 6:**
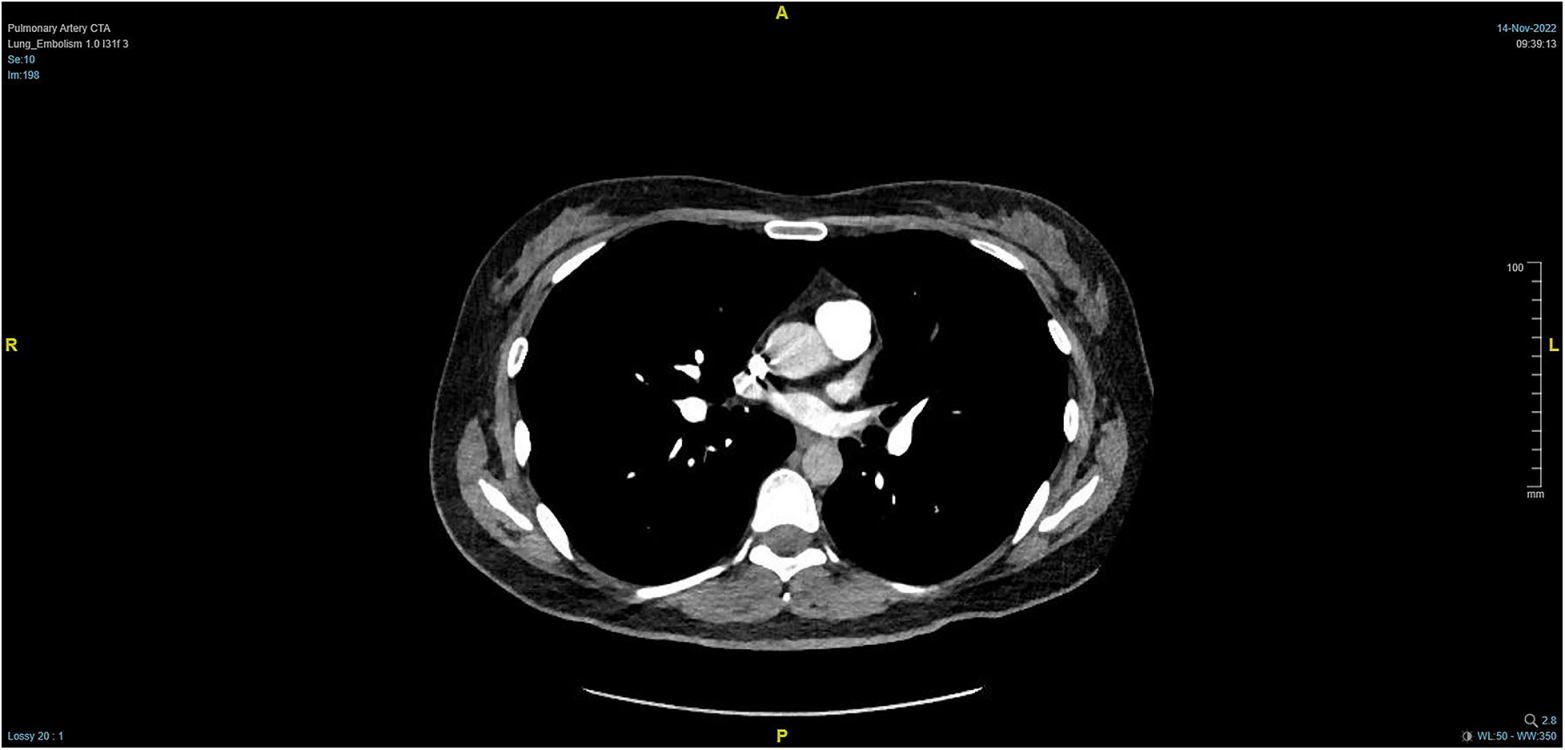
PTCA re-examination 2 months after discharge. Pulmonary embolism improved.

**Figure 7:**
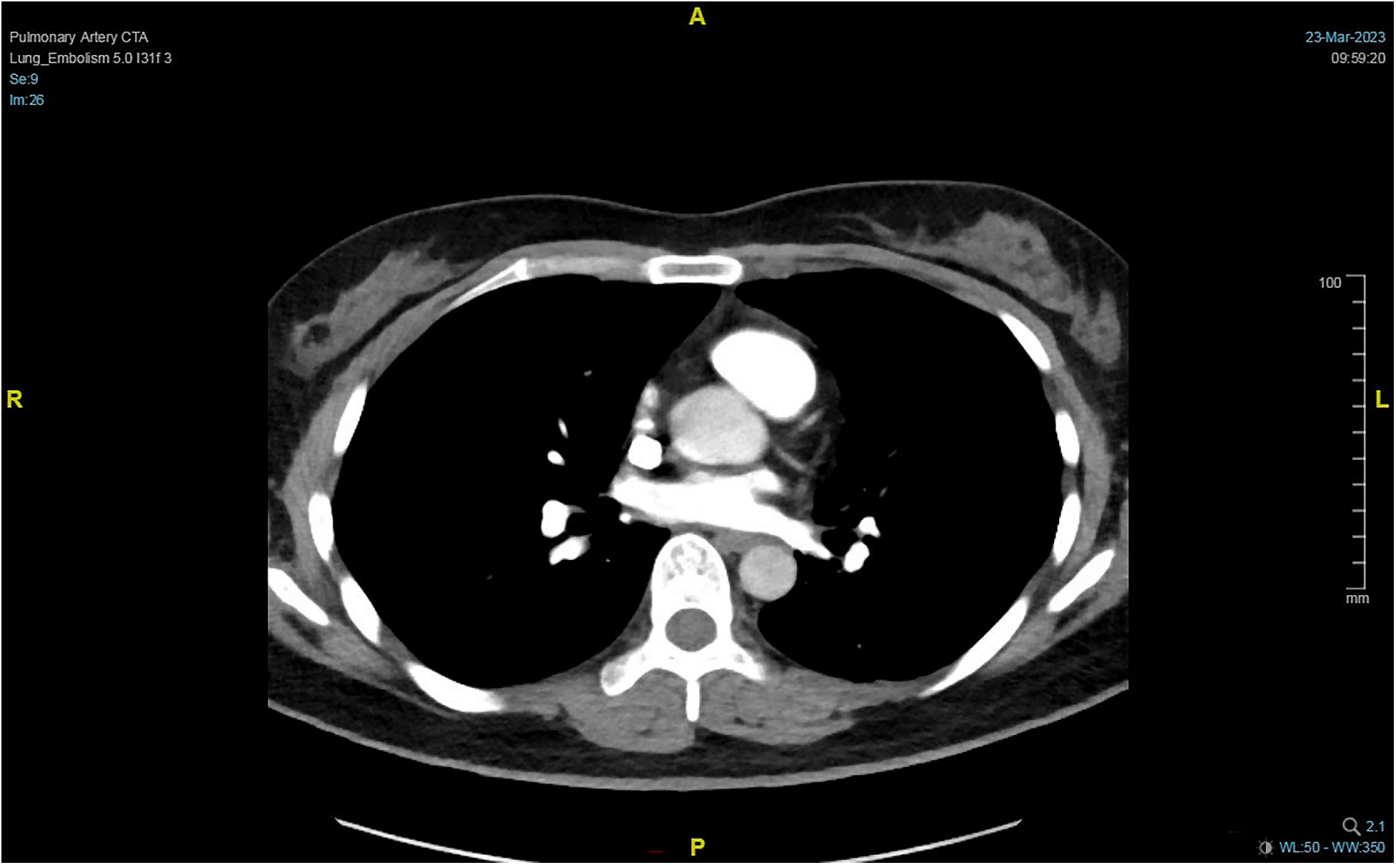
PTCA re-examination 7 months after discharge.

**Figure 8:**
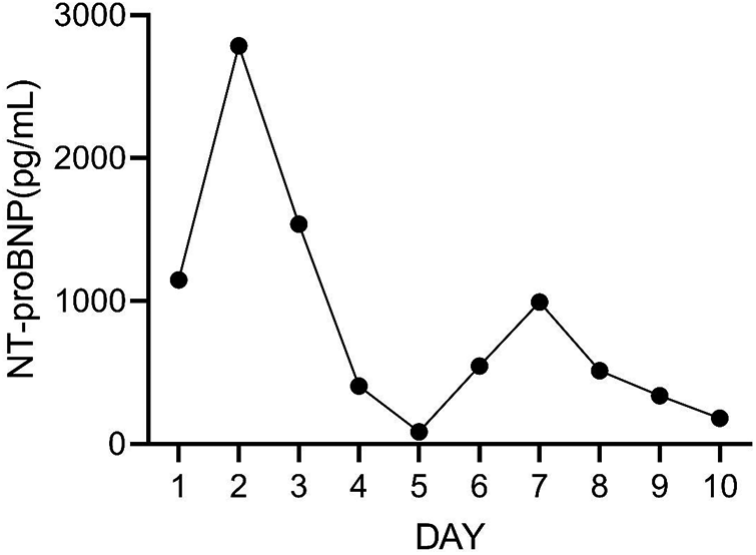
The concentration of NT-proBNP during the 10 days after admission.

**Figure 9:**
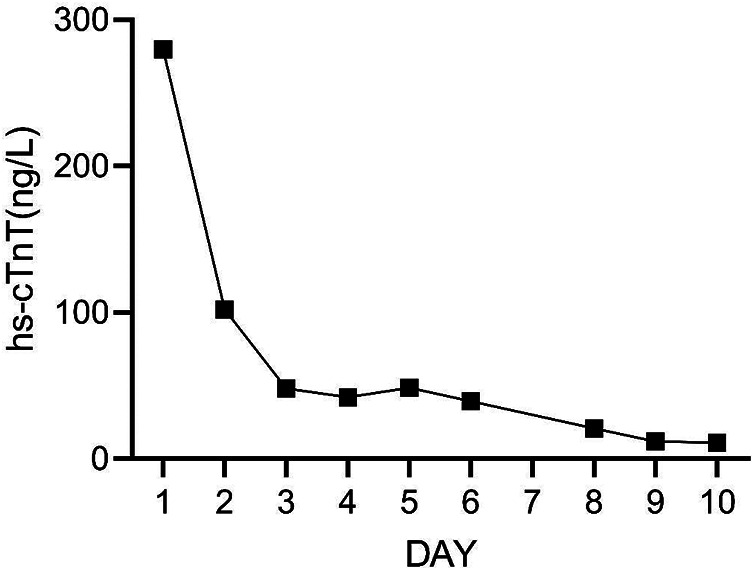
The concentration of hs-cTnT during the 10 days after admission.

**Figure 10:**
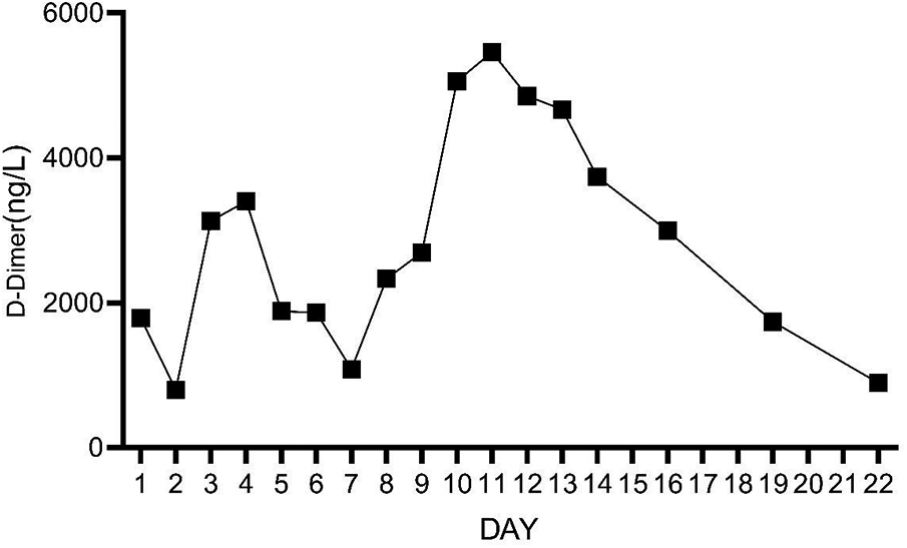
The concentration of D-Dimer during the 10 days after admission.

On Day 2 of VA-ECMO support, the hemodynamics and ventilation status were relatively stable, and “pump-controlled retrograde trial off (PCRTO)” was implemented to reduce the ECMO flow rate to −0.5 L/min—the end-tidal CO_2_ was 25 mmHg and the oxygen saturation of the right upper extremity was 100%. Bed side ultrasound was performed, and the results indicated obvious dilatation of the right ventricle, increased tricuspid regurgitation, and high pressure in the pulmonary artery. The patient had developed right ventricular dysfunction and increased pressure in the pulmonary artery. “PCRTO” was stopped immediately, and the flow rate was increased to 2.5 to 3.0 L/min. On Day 6 after the procedure, the PCRTO was successfully performed, leading to the discontinuation of VA-ECMO support. Three days after the patient was weaned off ECMO, their respiratory and circulatory functions stabilized resulting in the removal of the endotracheal tube, and continuous positive airway pressure was subsequently initiated. The patient was weaned of the continuous positive airway pressure on Day 4 after the procedure and was administered oxygen inhalation by bilateral nasal cannula, with good oxygenation index, and was transferred back to the general ward. The patient was administered continuous anticoagulation therapy with low molecular heparin and ambrisentan to lower pulmonary artery pressure, and other symptomatic treatment. On Day 22 after the procedure, the patient was discharged from the hospital. Post discharge, the patient continued to take anticoagulant drugs orally. Computed tomography pulmonary angiography re-examinations were conducted 2 and 7 months after discharge, and the results suggested that the pulmonary artery perfusion had improved, as shown in Figures 6, 7. We also monitored the indexes of NT-proBNP and hs-cTnT within 10 days and D-Dimer within 22 days of admission, and the data were shown in Figures 8–10.

## Discussion

Pulmonary embolism caused by tumor emboli is relatively rare in clinical practice, and there are no specific clinical manifestations. In fact, pulmonary embolism caused by tumor embolism is often more urgent and dangerous than pulmonary embolism caused by thrombus shedding. Generally, patients have not yet found malignant tumors, while cardiac arrest has been caused by tumor pulmonary embolism. However, conventional thrombolytic therapy has poor effect. Therefore, patients or whose family may present with complaints of the doctor due to symptoms of acute pulmonary embolism, however, the diagnosis is often confirmed only after pulmonary artery embolism is detected by autopsy. In patients with solid tumors, especially renal cell and hepatocellular carcinomas, pulmonary embolism caused by tumor emboli should be suspected when patients report to the emergency department with symptoms such as shortness of breath, hemoptysis, and chest pain (1, 2). What's more, secondary complex thromboembolism is highly likely to occur after tumor embolism. The thrombus stops the blood flow or forms eddy currents, leading to recurrent thrombosis, and may even become chronic pulmonary embolism, which can occur repeatedly and eventually lead to pulmonary hypertension. Tumor thrombus mixed with thrombus may lead to right heart enlargement, even right heart failure, and then affect the right heart and lead to total heart failure (3). In this case, pre-operative infiltration of inferior vena-cava was confirmed, and circulatory instability occurred during the intra-operative inferior vena-cava embolectomy; thus it was highly suspected that the tumor emboli were falling off and resulted in pulmonary embolism. The mortality rate of obstructive cardiogenic shock caused by massive pulmonary embolism is higher than 50%. VA-ECMO, as an effective resuscitation technique in an emergency, can effectively improve the prognosis and stabilize the patient's condition in a more ideal manner, such as reducing the right side heart load and improving the right side cardiac function and hemodynamic stability. For patients with severe respiratory and circulatory failure, VA-ECMO can effectively improve tissue perfusion, maintain the horizontal artery pressure above 60 mmHg, and stabilize circulation, giving patients a window of time to undergo surgery or other treatment for the primary disease. It also provides an opportunity for the patient to receive surgical or interventional embolectomy (4–6). ECMO is widely used in the rescue treatment of massive pulmonary embolism. ECPR mode is independently associated with mortality, and it is important to initiate ECPR before physiological deterioration or cardiac arrest occurs (7). Other studies have shown no significant difference in mortality between patients treated with ECMO for high-risk pulmonary embolism and those treated without ECMO. But it can lead to complications such as massive bleeding. Nevertheless, early use of ECMO may still be therapeutic for patients with indications. The next step is to continue to confirm the efficacy of ECMO in patients at high risk for pulmonary embolism through an extensive multicenter prospective trial (8). Standard treatment guideline recommended that low-grade ECMO support maybe associated with a survival benefit for patients (9). For patients with contraindications to thrombolysis and anticoagulation, VA-ECMO can be used as an effective cardiopulmonary aid to provide patients the opportunity for surgical or interventional embolectomy (10, 11). Anticoagulation therapy is not routinely recommended for tumor-induced pulmonary embolism, but it is still recommended for the prevention of secondary thrombosis (12, 13). With the ECMO technique being popular, an ECMO response team has been established in our hospital to provide immediate support in the case of life-threatening emergencies to improve hemodynamics, stabilize the condition and improve patient prognosis in the shortest time.

## Conclusion

Pulmonary embolism is a life-threatening condition, and tumor-induced pulmonary embolism is rare in clinical practices. Early detection and timely surgical or interventional embolectomy is still the primary treatment method. However, most of the patients experience progressive respiratory and circulatory failure after the onset of the disease, preventing them from receiving early treatment. VA-ECMO provides the opportunity for timely surgical and interventional embolectomy, improving the short-and long-term prognosis.

## Data Availability

The original contributions presented in the study are included in the article/Supplementary Material, further inquiries can be directed to the corresponding author.
