# Supplementary figures and images for: Case Report: Resuscitation of patient with tumor-induced acute pulmonary embolism by venoarterial extracorporeal membrane oxygenation
Source: Front Cardiovasc Med. 2024 Feb 15;11:1322387. doi: 10.3389/fcvm.2024.1322387 (PMC10902156; doi:10.3389/fcvm.2024.1322387)

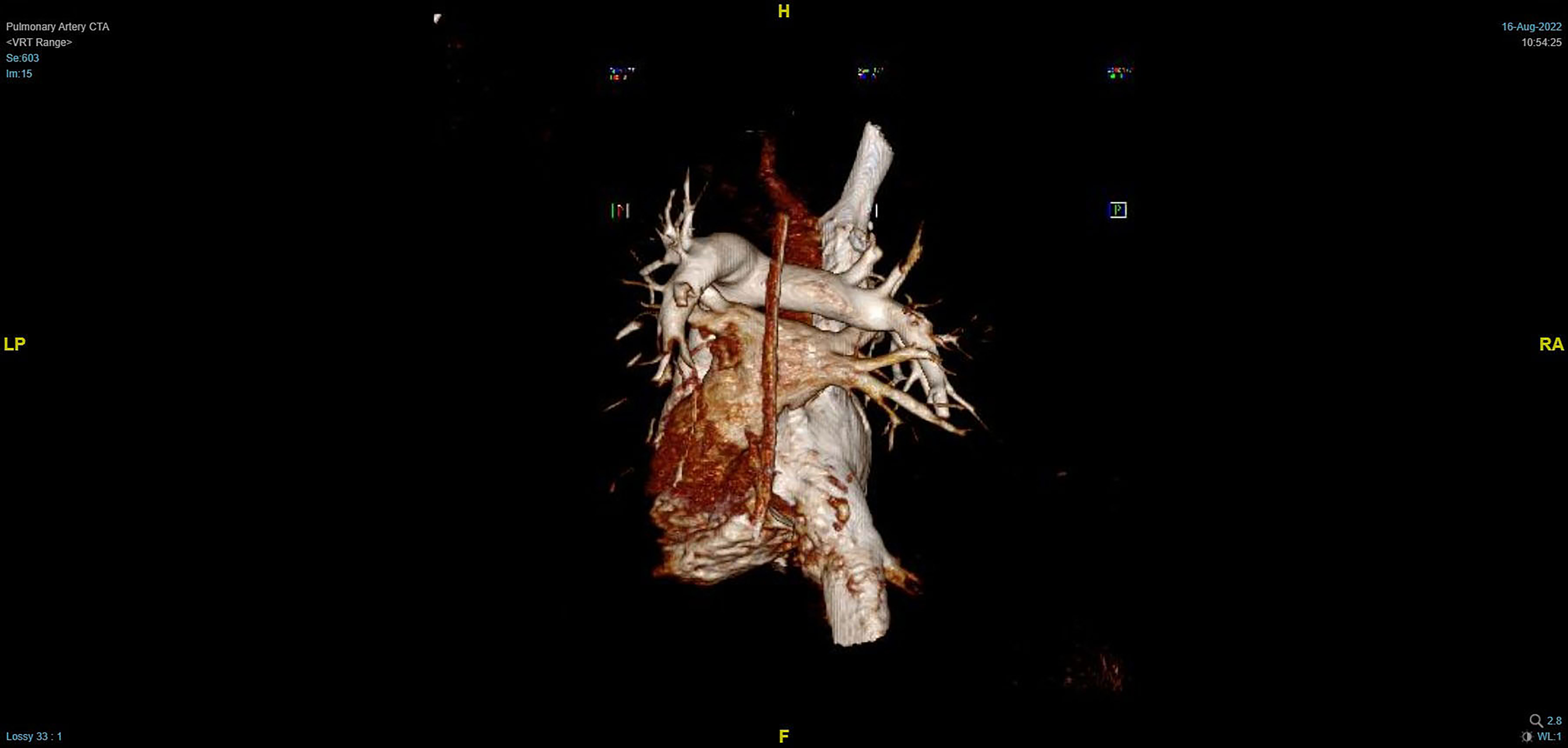

Supplement: Supplementary file 1 [file Image1.jpeg]

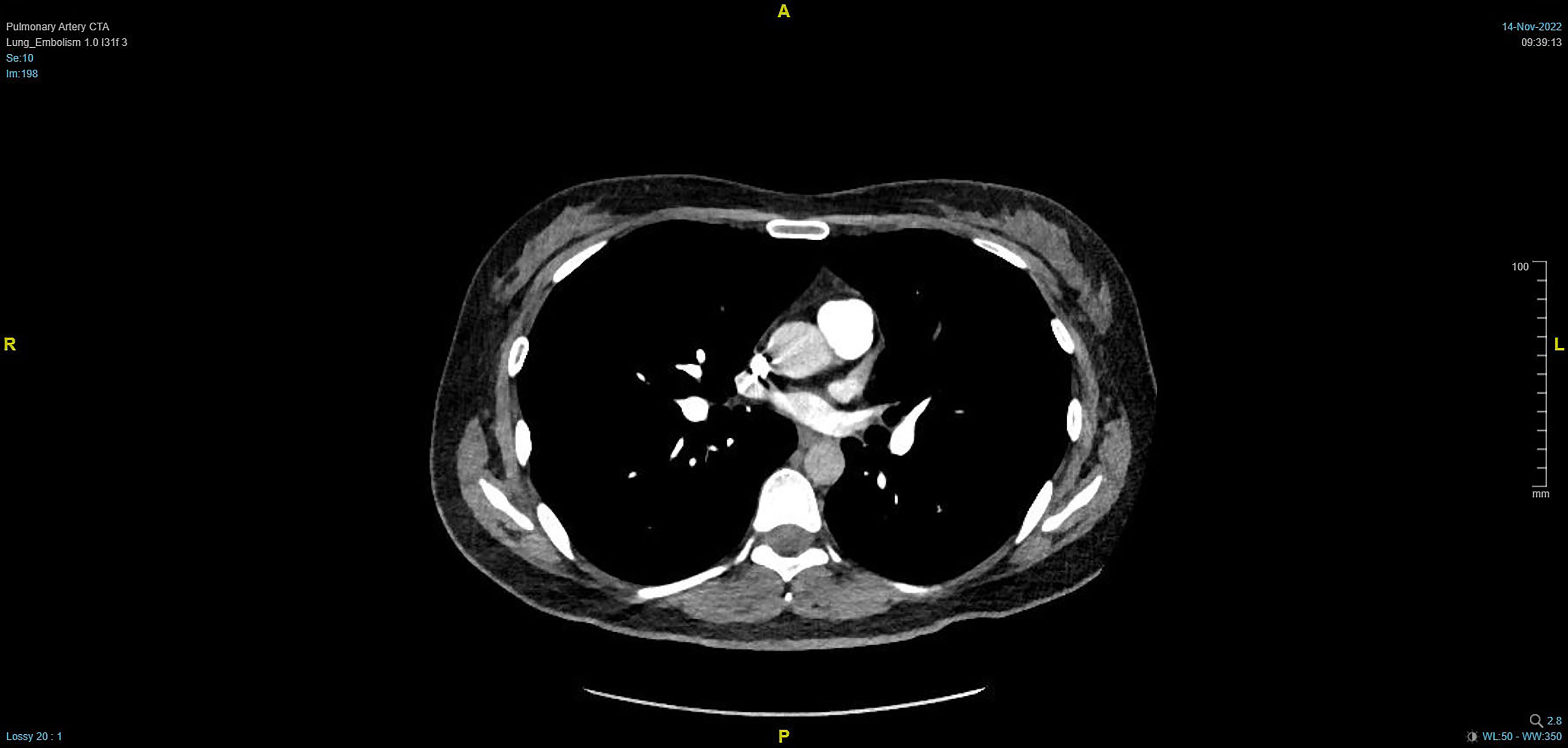

Supplement: Supplementary file 2 [file Image2.jpeg]

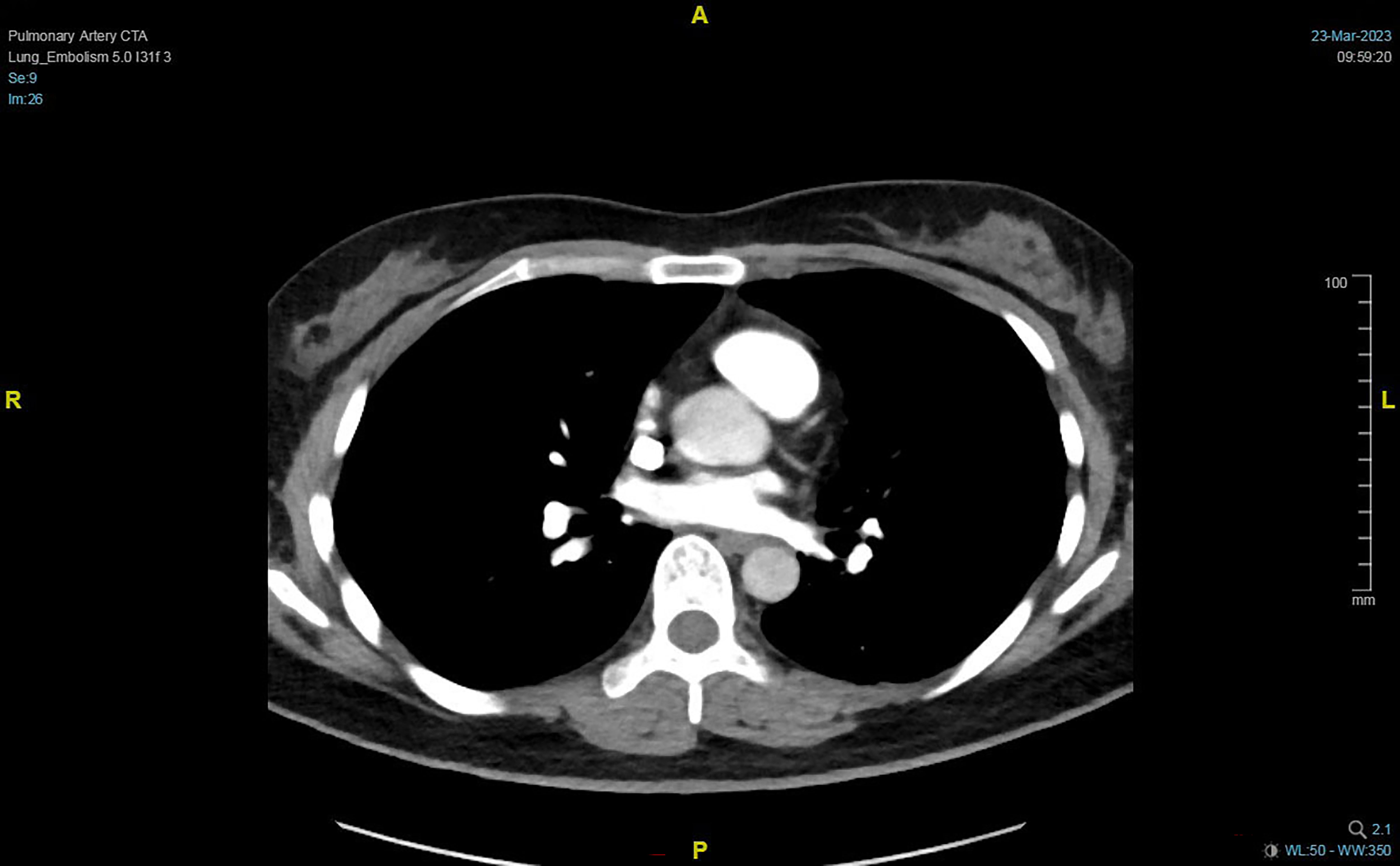

Supplement: Supplementary file 3 [file Image3.jpeg]

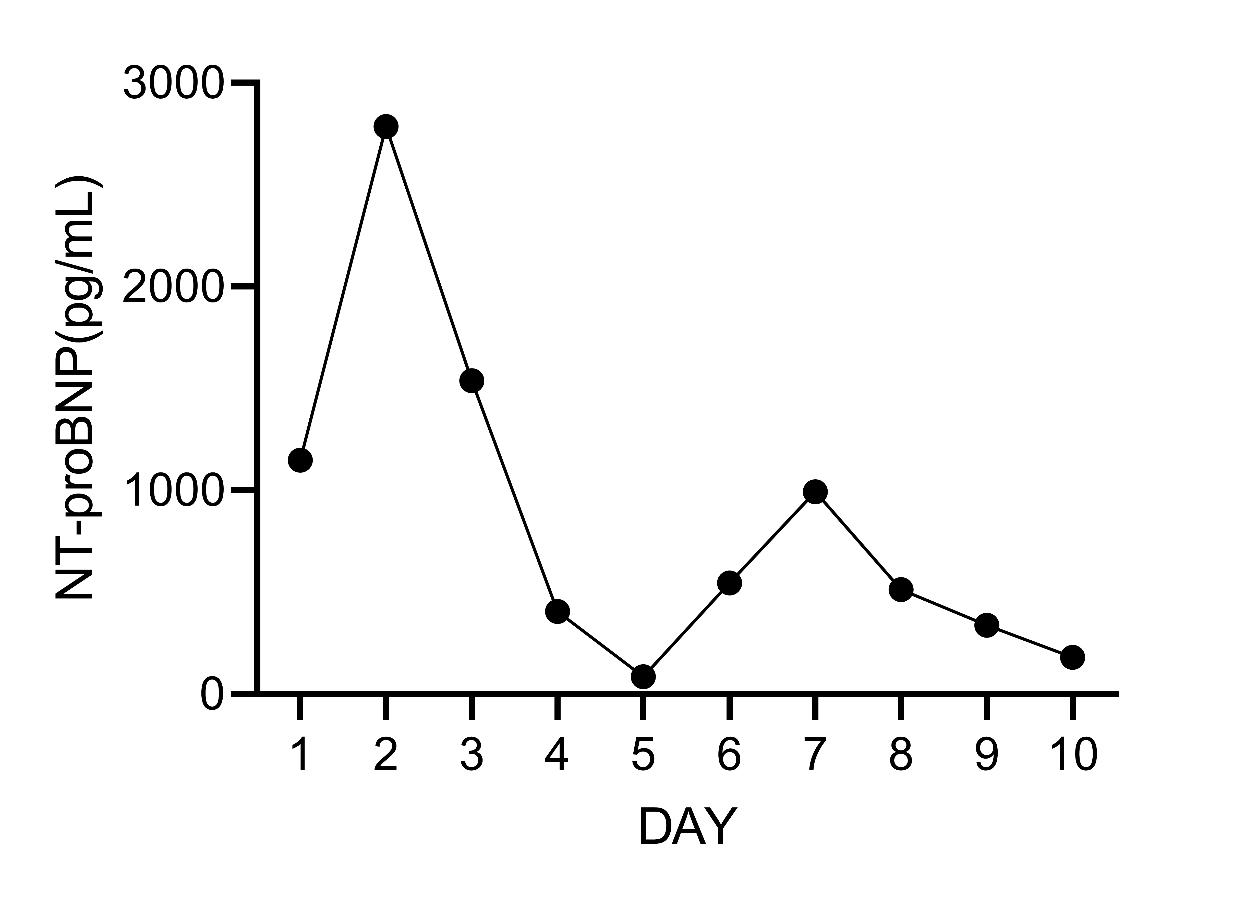

Supplement: Supplementary file 4 [file Image4.jpeg]

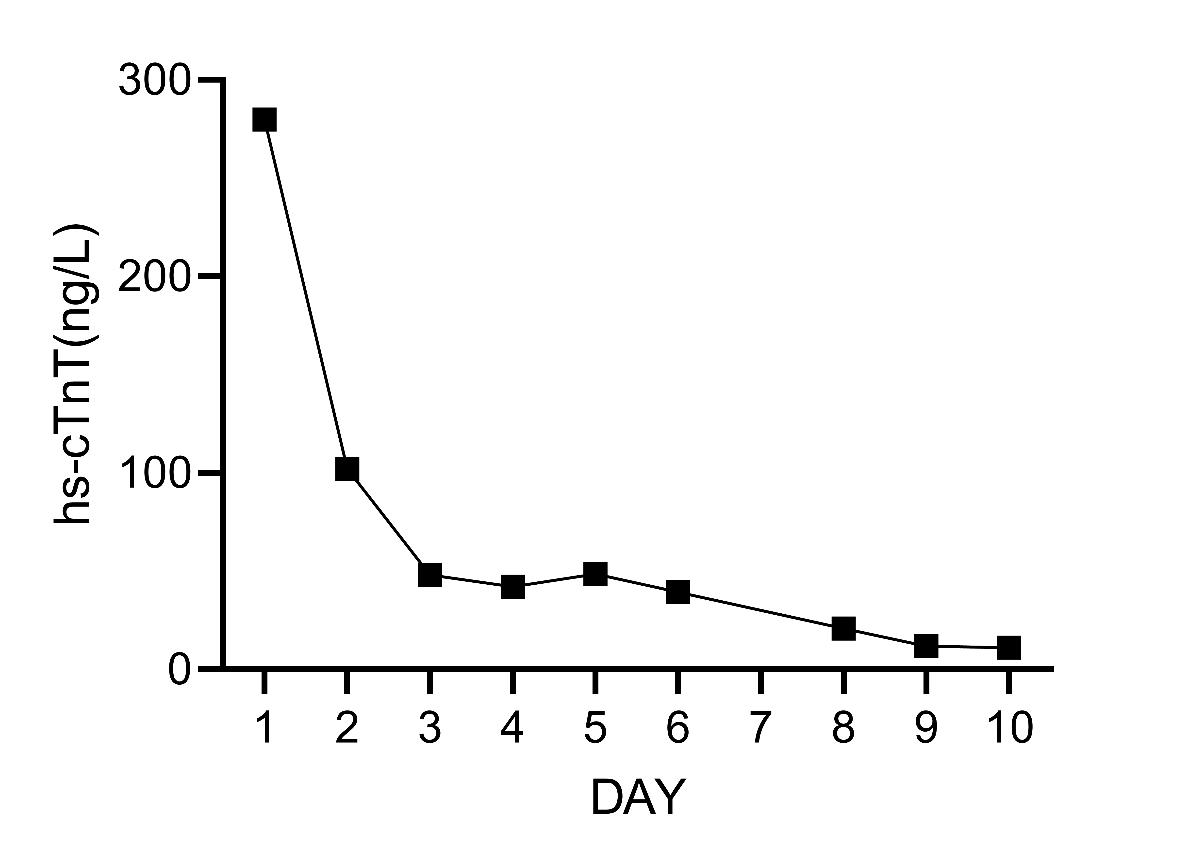

Supplement: Supplementary file 5 [file Image5.jpeg]

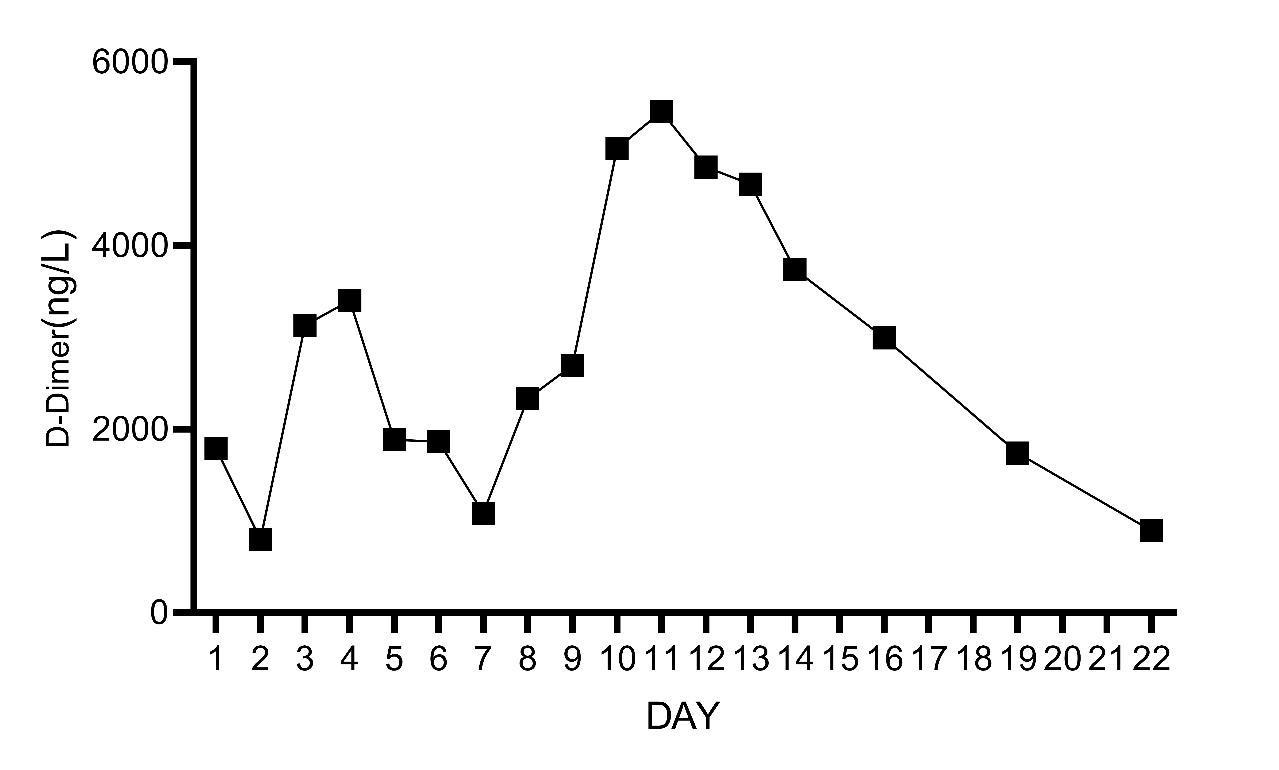

Supplement: Supplementary file 6 [file Image6.jpeg]
